# Impact of the COVID-19 Pandemic on Changes in Tobacco Use Behavior: A Longitudinal Cohort Study in Japan

**DOI:** 10.2188/jea.JE20240180

**Published:** 2025-06-05

**Authors:** Makiko Kanai, Osamu Kanai, Takahiro Tabuchi

**Affiliations:** 1 Division of Respiratory Medicine, National Hospital Organization Kyoto Medical Center, Kyoto, Japan; 2 Department of Cancer Epidemiology, Osaka International Cancer Institute Cancer Control Center, Osaka, Japan

**Keywords:** tobacco cessation, heated tobacco products, COVID-19 pandemic

## Abstract

**Background:**

Use of novel tobacco products, such as heated tobacco products, has recently increased as a result of being promoted less harmful alternatives to cigarettes. The impact of the coronavirus disease 2019 (COVID-19) pandemic on tobacco use may differ depending on the type of tobacco.

**Methods:**

We longitudinally investigated changes in tobacco use over a 1-year period using internet-based and self-reported questionnaires among Japanese aged 15 to 79 years. The study was conducted from 2019 to 2021, with participants before the COVID-19 pandemic in February 2020 as the pre-pandemic group and participants after that as the pandemic group. Accounting for population bias, we used sampling probability weighting referring to the nationwide data. The association between cessation and the COVID-19 pandemic was evaluated separately for each type of tobacco using logistic regression analysis.

**Results:**

After conducting sampling probability weighting, 1,920 were in the pre-pandemic group and 2,681 were in the pandemic group. More participants in the pandemic group than in the pre-pandemic group achieved cessation after 1 year (13.8% vs 10.2%, *P* < 0.001). Dual users were more likely to quit during the pandemic than pre-pandemic (adjusted odds ratio [aOR] 2.56, *P* < 0.001), whereas exclusive novel tobacco users were less likely to quit during the pandemic (aOR 0.66, *P* = 0.041). Tobacco cessation was more frequently achieved among those who had intended to quit at baseline survey among conventional tobacco users (aOR 1.77, *P* < 0.001) and dual users (aOR 2.52, *P* < 0.001); however, this trend was not observed among novel tobacco users (aOR 1.49, *P* = 0.090).

**Conclusion:**

Conventional and novel tobacco use patterns varied in response to the COVID-19 pandemic.

## INTRODUCTION

Tobacco smoking is the leading preventable cause of death worldwide, and quitting tobacco is a major and immediate health benefit for people of all ages.^1^^–^^3^ Knowledge of the adverse health effects of tobacco use can motivate people to quit tobacco, with approximately two-thirds of cigarette smokers reported to be interested in quitting.^3^^,^^4^ However, nicotine contained in tobacco products is highly addictive, and only 4% of users who attempt to quit tobacco use will succeed without cessation support.^1^^,^^3^^,^^4^

In recent years, novel tobacco products, such as e-cigarettes and heated tobacco products (HTPs), have been promoted as reduced-harm products or products that can help people quit combustible tobacco smoking.^1^ The tobacco industry’s campaign encourages people “who don’t quit cigarettes” to “change to a better alternative”.^5^ Since the launch of HTPs in Japan, the prevalence of HTP use has increased explosively from 0.2% in 2015 to 11.3% in 2019, and Japan has become the largest market for HTPs worldwide.^6^^,^^7^ On the other hand, the consumption of cigarettes in Japan is decreasing yearly.^8^ According to the Tobacco Institute of Japan, domestic sales in fiscal year 2020 fell 16.3% from the previous fiscal year, the largest drop since fiscal year 1990 for which comparable data are available.^9^ The coronavirus disease 2019 (COVID-19) pandemic emerged in late 2019 and spread rapidly to other countries across the world. This led World Health Organization to declare a Public Health Emergency of International Concern and governments implemented a range of restrictions, including lockdowns and social distancing measures. Most smokers believed that smoking increased COVID-19 risk and this large drop may have resulted from the behavior changes in cigarette users driven by the COVID-19 pandemic.^10^^,^^11^ In contrast to the decline in cigarette consumption, the share of HTP sales increased from 23.5% in 2019 to 29.5% in 2020.^9^ In short, changes in tobacco product sales before and during the COVID-19 pandemic differed by the type of tobacco product.

We hypothesized that the impact of the COVID-19 pandemic on tobacco use behavior may have differed depending on the type of tobacco product used. In this study, we longitudinally investigated tobacco usage patterns and compared tobacco use statuses between baseline and the 1-year follow-up to understand the association more clearly between COVID-19 and tobacco use. Furthermore, we investigated whether there was a difference in behavioral changes depending on participants’ living environment, intention to quit, health status, and subjective happiness to evaluate trends in tobacco cessation during the COVID-19 pandemic.

## METHODS

### Study subjects and design

We conducted an analysis of data obtained from two internet-based, self-report questionnaire surveys: the Japan Society and New Tobacco Internet Survey (JASTIS) and the Japan COVID-19 and Society Internet Survey (JACSIS).^12^^,^^13^ These surveys were administered by a prominent internet research agency, Rakuten Insight, Inc., which boasts a large panel of approximately 2.3 million individuals.^14^ Registered individuals are assured through annual updates of demographic information and the exclusion of individuals with concerns about incorrect information. The surveys collect demographic, health-related, and socioeconomic information from individuals aged 15 to 79 years. The questionnaires were distributed to individuals selected by sex, age, and prefecture category using simple random sampling and covering all 47 prefectures (first-tier administrative districts in Japan). Individuals who consented to participate in the survey visited the designated website and completed the survey. They also had the option not to respond or to discontinue at any point during the survey. In such cases, they were regarded as not having consented to participate in the survey and were not counted as respondents. Questionnaires were distributed until the target number of respondents for each sex, age, and prefecture category were met, which was determined based on the Japanese census. To validate the quality of the data, we excluded responses with discrepancies and/or unnatural responses. The following exclusion criteria were used: (1) an invalid response to “Please choose the second option from the bottom” (2), positive responses to all questions related to drug use (eg, marijuana, cocaine, or heroin), and (3) positive responses to all questions regarding the underlying 16 alternative chronic diseases. In this study, we included those who participated in both the JASTIS 2019 (February 2019) and JASTIS 2020 (February 2020) in the pre-pandemic group. We included in the pandemic group those who had newly participated in both the JASTIS 2020 and JASTIS 2021 (February 2021) or both the JACSIS 2020 (September 2020) and JACSIS 2021 (September 2021).

### Ethical issues

All procedures were conducted in accordance with the ethical standards of the Declaration of Helsinki of 1975, as revised in 2013. The study protocol was reviewed and approved by the Research Ethics Committee of the Osaka International Cancer Institute (approval no. 1412175183, 20084). The internet survey agency respected the Act on the Protection of Personal Information in Japan. All participants provided web-based informed consent before responding to the online questionnaire.

### Measures

#### Sociodemographic information

Sociodemographic variables included age, sex, education (higher education defined as bachelor’s degree or higher vs no degree), occupation (employed vs unemployed), living status (living alone vs living with cohabitant), annual household income, and marital status (married vs not married).

#### Health conditions

Health conditions included overweight or obesity, defined as a body mass index (BMI) ≥25 kg/m^2^, habitual alcohol intake (drink within 30 days vs no drink within 30 days), and comorbidities. Comorbidities included respiratory diseases (asthma, chronic obstructive pulmonary disease, and bronchitis), diabetes mellitus, hypertension, and mental disorders; these were answered yes/no. Self-reported happiness was rated directly on a scale of 1 to 10, with a score of 10 indicating the highest level of happiness. Self-reported health status was subjectively classified into five categories, with a score of 1 indicating good and 5 indicating poor self-rated health status.

#### Tobacco-related questions

Tobacco use status was asked for each tobacco product: conventional cigarettes (cigarette, hand-rolled tobacco, cigar, little cigar, pipe tobacco), HTP, and e-cigarette. We defined HTP and e-cigarette as novel tobacco because e-cigarettes are not as popular as HTP in Japan and the two are often confused. We defined current tobacco use as using any tobacco product in the last 30 days. We classified tobacco status into three categories: exclusive conventional tobacco use, dual use (both conventional and novel tobacco), and exclusive novel tobacco use. Tobacco cessation was defined as quitting all kinds of tobacco in the past 30 days at the time of 1-year follow-up survey.

#### Outcome

The primary outcome was the incidence of tobacco cessation at the follow-up survey performed 1 year later. Secondary outcomes were changes in the use of each tobacco product and associated factors for tobacco cessation for each tobacco use status.

### Statistical analysis

Baseline characteristics of the participants and tobacco use status were summarized as numbers and percentages for categorical variables and means and standard deviations (SDs) for continuous variables. We compared the pre-pandemic and pandemic groups to examine the impact of the COVID-19 pandemic on tobacco use status. We used the chi-square test for categorical variables and the *t* test for continuous variables in univariable analyses. We used logistic regression analysis to calculate the odds ratios (ORs) of quitting each tobacco product in univariable and multivariable analyses. The univariable analysis regarding changes in the use of each tobacco product was performed only among participants who were candidates for changes in tobacco use status. In the multivariable analysis of model 1, ORs were adjusted by the following baseline characteristics of the participants: age, sex, overweight or obesity, alcohol intake, comorbidities, self-reported happiness, health status, concern about quitting tobacco products, education, occupation, and living status. Interactions between the groups and tobacco use status with exclusive conventional tobacco use as a reference were estimated in model 2 to assess the difference in the impact of the COVID-19 pandemic across tobacco use statuses in the baseline survey. Accounting for population bias due to the naturality of internet surveys, we used sampling probability weighting in all analyses. To estimate the sampling probability, we referred to the existing nationwide data from the Comprehensive Survey of Living Conditions performed by the Ministry of Health, Labour and Welfare in Japan. The tests were two-tailed, and the alpha error was set at 0.05. All statistical analyses were performed by IBM SPSS Statistics for Windows, Version 28.0 (IBM Corp., Armonk, NY, USA), and Sankey diagrams for the change in tobacco use status were generated by R version 4.2.2 with the tidyverse and networkD3 packages (R Foundation for Statistical Computing, Vienna, Austria).

## RESULTS

**Figure 1.**
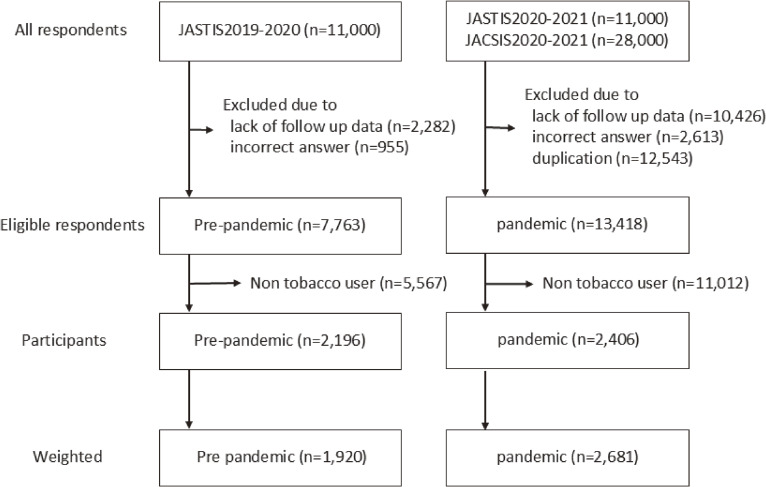
Participant selection and study flowchart from the JASTIS and JACSIS. The study flowchart shows the number of participants in each exclusion and weighting step.

**Figure 2.**
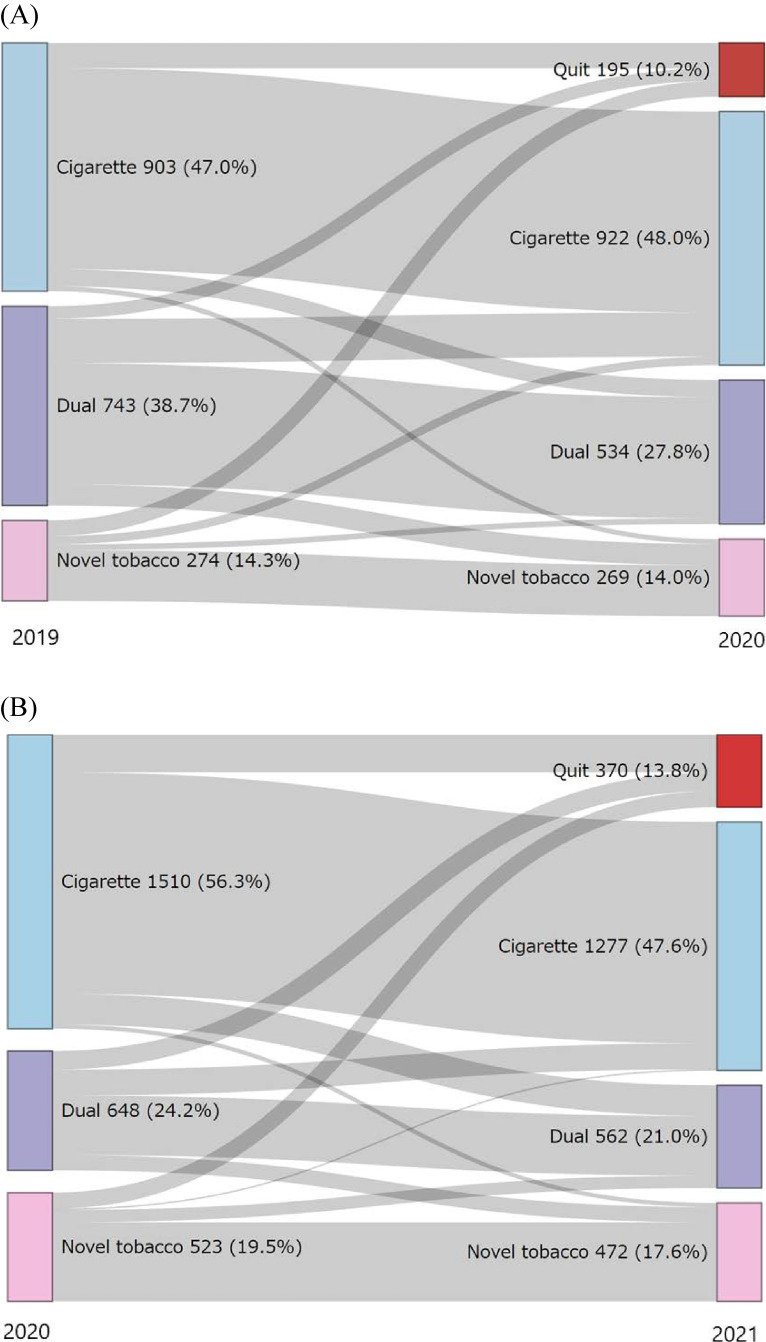
Changes in tobacco use status before and during the pandemic by sampling probability weighting. These Sankey diagrams show the changes in tobacco use status before (**A**) and during (**B**) the pandemic. The source nodes represent baseline tobacco use status, and the target nodes represent tobacco use status after 1 year. Each node is marked with the following colors: exclusive cigarette use is marked with blue, dual use is marked with purple, and novel tobacco product use is marked with pink. The width of the flow is proportional to the amount of flow.

After excluding individuals with missing follow-up data, incorrect answers, and duplicate questionnaires, 7,763 participants in the pre-pandemic group and 13,418 participants in the pandemic group were eligible for further analysis. Among them, 2,196 tobacco users in the pre-pandemic group and 2,406 tobacco users in the pandemic group were included in the final analysis (Figure 1). Table 1 shows the baseline characteristics of the participants corrected by sampling probability weighting. The unweighted baseline characteristics of the participants are shown in eTable 1. The proportion of females in the pandemic group was significantly higher. The proportions of people with habitual alcohol intake, concern about quitting tobacco, and who worked outside the home were significantly lower than those in the pre-pandemic group. People with exclusive conventional tobacco use, dual use, and exclusive novel tobacco product use were 1,510 (56.3%) versus 903 (47.0%), 648 (24.2%) versus 743 (38.7%), and 523 (19.5%) versus 274 (14.3%) in the pandemic and pre-pandemic groups, respectively. The changes in tobacco use status from the previous year by the type of tobacco are shown in Figure 2. The unweighted changes in tobacco use status by the type of tobacco are shown in eFigure 1 and eFigure 2.

**Table 1.** Baseline characteristics of the participants (weighted)

	Group	Pre-pandemic		Pandemic		*P* value	SMD
Number		1,920		2,681			
Baseline survey	JASTIS 2019	1,920	(100.0)	0	0.0	<0.001	2.179
	JASTIS 2020	0	0.0	216	(8.1)		
	JACSIS 2020	0	0.0	2,465	(91.9)		
Age, years	20s	106	(5.5)	260	(9.7)	0.383	
	30s	264	(13.7)	387	(14.4)		
	40s	525	(27.3)	672	(25.1)		
	50s	591	(30.8)	629	(23.4)		
	60s	384	(20.0)	457	(17.1)		
	70s	50	(2.6)	277	(10.3)		
Sex	Female	358	(18.6)	832	(31.0)	<0.001	0.29
BMI, kg/m^2^		23.1	[3.6]	22.9	[3.8]	0.253	0.054
Overweight or obesity^a^		530	(27.6)	647	(24.1)	0.099	0.08
Habitual alcohol intake		1,300	(67.7)	1,619	(60.4)	0.002	0.152
Hypertension		384	(20.0)	553	(20.6)	0.735	0.016
Diabetes mellitus		158	(8.2)	237	(8.9)	0.622	0.023
Chronic respiratory diseases		124	(6.5)	163	(6.1)	0.740	0.017
Mental disorders		166	(8.6)	216	(8.0)	0.657	0.022
Self-reported health status^b^		2.7	[0.9]	2.6	[1.0]	0.035	0.095
Self-reported happiness^c^		6.1	[2.3]	6.5	[2.2]	0.988	
Concern about quitting tobacco		1,300	(67.7)	1,715	(63.9)	0.009	
Education	Beyond high school	707	(36.8)	764	(28.5)	<0.001	0.178
Working outside the home		1,573	(81.9)	2,072	(77.3)	0.019	0.114
Annual household income	1st quartile	500	(26.0)	775	(28.9)	0.321	0.105
	2nd quartile	412	(21.4)	565	(21.1)		
	3rd quartile	417	(21.7)	500	(18.6)		
	4th quartile	288	(15.0)	367	(13.7)		
	Unknown	305	(15.9)	476	(17.7)		
Number of cohabitants		2.9	(2.7)	3.0	(3.1)	0.504	0.027
Living alone		338	(17.6)	431	(16.1)	0.367	0.041
Married		1,270	(66.1)	1,730	(64.5)	0.479	0.034

BMI, body mass index; SMD, standardized mean difference.

Data are shown with numbers and (percentages) or means and [standard deviations]. Differences between the groups are evaluated by the SMD. *P* values were estimated by chi-square tests for categorical variables and by *t* tests for continuous variables.

^a^Overweight or obesity was defined as 25 kg/m^2^ or over.

^b^Self-reported health status was evaluated by 5 rank scores: 1 (excellent), 2 (good), 3 (moderate), 4 (poor), and 5 (awful).

^c^Self-reported happiness was evaluated by 10 rank scores: 1 (awful) to 10 (excellent).

After 1 year of follow-up, significantly more participants in the pandemic group achieved tobacco cessation than in the pre-pandemic group (13.8% vs 10.2%, *P* < 0.001) (OR 1.42, *P* < 0.001) (Table 2 and eTable 2).

**Table 2.** Odds ratios for the pandemic group with pre-pandemic group as the reference were estimated by logistic regression analysis without adjustment (weighted)

		OR	95% CI	*P* value
Quit all tobacco products		1.42	(1.18–1.71)	<0.001
Conventional tobacco products	Quit	1.37	(1.15–1.63)	<0.001
	Started	0.69	(0.46–1.02)	0.060
Novel tobacco products	Quit	0.92	(0.76–1.10)	0.355
	Started	1.39	(1.06–1.84)	0.019

CI, confidence interval; OR, odds ratio.

When analyzed by type of tobacco, the proportion of people who quit conventional tobacco products was significantly higher in the pandemic group than in the pre-pandemic group (18.6% vs 14.3%, *P* < 0.001) (OR 1.37, *P* < 0.001). In contrast, no significant differences were observed in the proportion of people who quit novel tobacco products (27.1% vs 28.9%, *P* = 0.558) (OR 0.92, *P* = 0.355). The proportion of new initiations or resumptions of novel tobacco products was significantly higher in the pandemic group than in the pre-pandemic group (OR 1.39, *P* = 0.019). The results of unweighted analysis for ORs are shown in eTable 3 and eTable 4.

Table 3 shows the weighted results of the multivariable logistic regression analysis of quitting all tobacco products among all participants. In model 1, tobacco cessation was improved after the COVID-19 pandemic (adjusted OR [aOR] 1.32, *P* = 0.005). Moreover, there was a significant interaction between the COVID-19 pandemic and baseline tobacco use status in model 2. Due to the significant interaction, we analyzed the data separately by baseline tobacco use status. A significant change in tobacco cessation during the COVID-19 pandemic was not observed among exclusive conventional tobacco product users (aOR 1.24, *P* = 0.121) (Table 4). The proportion of tobacco cessation significantly increased after the COVID-19 pandemic among dual users (aOR 2.56, *P* < 0.001) (Table 4). In contrast, the proportion of tobacco cessation significantly decreased during the COVID-19 pandemic among exclusive novel tobacco product users (aOR 0.66, *P* = 0.041) (Table 4). Tobacco cessation was more frequently achieved among those who had the intention to quit at the time of baseline survey among exclusive conventional tobacco product users (aOR 1.77, *P* < 0.001) and dual users (aOR 2.52, *P* < 0.001); however, tobacco cessation was not associated with the intention to quit among exclusive novel tobacco product users (aOR 1.49, *P* = 0.090) (Table 5A, Table 5B, and Table 5C).

**Table 3.** Multivariable analysis for tobacco cessation among all participants (weighted)

		Model 1			Model 2		
		aOR^a^	95% CI	*P* value	aOR^a^	95% CI	*P* value
Pandemic		1.32	(1.09–1.60)	0.005	1.21	(0.92–1.58)	0.168
Baseline tobacco use status	exclusive conventional tobacco users	1.00			1.00		
	dual users	0.83	(0.67–1.04)	0.110	0.54	(0.37–0.78)	0.001
	exclusive novel tobacco users	1.32	(1.05–1.65)	0.018	1.80	(1.24–2.61)	0.002
Pandemic * (dual users)					2.09	(1.32–3.32)	0.002
Pandemic * (exclusive novel tobacco users)					0.62	(0.39–0.99)	0.045

aOR, adjusted odds ratio; CI, confidence interval.

^a^Adjusted odds ratios for quitting all tobacco products were estimated by the logistic regression analysis. In model 1, aORs were adjusted for age, sex, overweight or obesity, alcohol intake, comorbidities, self-reported happiness and health status, concern about quitting tobacco products, education, occupation, and living status. To assess differences in the impact of the COVID-19 pandemic across tobacco use statuses at baseline, interaction terms between pandemic and tobacco use status were added in model 2. “Pandemic * (dual users)” and “Pandemic * (exclusive novel tobacco users)” indicate the interaction terms between the groups and dual use and between the groups and exclusive novel tobacco users. In the interaction terms, exclusive conventional tobacco product use is treated as a reference for baseline tobacco use status.

**Table 4.** Adjusted odds ratios of the effect of the pandemic on tobacco cessation among participants with each tobacco use status

	aOR	95% CI	*P* value
Exclusive conventional tobacco product users	1.24	(0.94–1.63)	0.121
Dual users	2.56	(1.73–3.79)	<0.001
Exclusive novel tobacco product users	0.66	(0.44–0.98)	0.041

aOR, adjusted odds ratio; CI, confidence interval.

Odds ratios for quitting all tobacco products were estimated for each tobacco use status in the baseline survey by logistic regression analysis. In each analysis, ORs were adjusted for age, sex, overweight or obesity, alcohol intake, comorbidities, self-reported happiness and health status, concern about quitting tobacco products, education, occupation, and living status.

**Table 5A.** Adjusted odds ratios for tobacco cessation among exclusive cigarette users

	aOR	95% CI	*P* value
Pandemic	1.24	(0.94–1.63)	0.121
Age^a^ 40–59 years^2^	0.36	(0.26–0.49)	<0.001
Age^a^ ≥60 years	0.32	(0.22–0.48)	<0.001
Sex, female	1.56	(1.17–2.09)	0.003
Overweight or obesity	1.45	(1.08–1.94)	0.014
Habitual alcohol intake	1.35	(1.02–1.77)	0.034
Hypertension	1.16	(0.82–1.65)	0.393
Diabetes mellitus	1.39	(0.87–2.22)	0.165
Chronic respiratory diseases	0.40	(0.19–0.89)	0.024
Mental disorders	1.07	(0.65–1.74)	0.793
Bad health status	1.10	(0.73–1.66)	0.633
Feeling happy	1.41	(1.05–1.91)	0.023
Having concern for quitting	1.77	(1.33–2.36)	<0.001
Higher education	1.29	(0.98–1.69)	0.070
Working outside the home	0.92	(0.66–1.29)	0.642
Living with cohabitants	1.21	(0.84–1.73)	0.299

aOR, adjusted odds ratio; CI, confidence interval.

Adjusted odds ratios for quitting all tobacco products are estimated by the logistic regression analysis.

^a^Age reference group: 20–39 years.

**Table 5B.** Adjusted odds ratios for tobacco cessation among dual users

	aOR	95% CI	*P* value
Pandemic	2.56	(1.73–3.79)	<0.001
Age^a^ 40–59 years^2^	0.50	(0.32–0.78)	0.002
Age^a^ ≥60 years	0.55	(0.30–1.02)	0.059
Sex, female	0.88	(0.55–1.39)	0.575
Overweight or obesity	0.44	(0.26–0.74)	0.002
Habitual alcohol intake	0.54	(0.37–0.79)	0.002
Hypertension	0.77	(0.44–1.35)	0.362
Diabetes mellitus	1.67	(0.90–3.13)	0.106
Chronic respiratory diseases	0.77	(0.39–1.52)	0.451
Mental disorders	1.23	(0.69–2.20)	0.486
Bad health status	1.23	(0.68–2.21)	0.493
Feeling happy	1.15	(0.76–1.73)	0.510
Having concern for quitting	2.52	(1.57–4.05)	<0.001
Higher education	1.25	(0.84–1.86)	0.265
Working outside the home	0.64	(0.36–1.13)	0.120
Living with cohabitants	0.72	(0.46–1.14)	0.165

aOR, adjusted odds ratio; CI, confidence interval.

Adjusted odds ratios for quitting all tobacco products are estimated by the logistic regression analysis.

^a^Age reference group: 20–39 years.

**Table 5C.** Adjusted odds ratios for quitting all tobacco products among exclusive novel tobacco users

	aOR	95% CI	*P* value
Pandemic	0.66	(0.44–0.98)	0.041
Age^a^ 40–59 years	0.39	(0.25–0.61)	<0.001
Age^a^ ≥60 years	1.08	(0.58–2.01)	0.802
Sex, female	1.23	(0.79–1.91)	0.367
Overweight or obesity	0.54	(0.32–0.91)	0.021
Habitual alcohol intake	2.03	(1.28–3.23)	0.003
Hypertension	1.28	(0.76–2.15)	0.360
Diabetes mellitus	0.54	(0.21–1.44)	0.219
Chronic respiratory diseases	2.79	(1.37–5.70)	0.005
Mental disorders	0.81	(0.32–2.08)	0.667
Bad health status	1.04	(0.54–2.03)	0.898
Feeling happy	0.78	(0.50–1.22)	0.278
Having concern for quitting	1.49	(0.94–2.37)	0.090
Higher education	1.11	(0.73–1.68)	0.631
Working outside the home	0.67	(0.39–1.17)	0.160
Living with cohabitants	1.72	(0.88–3.35)	0.112

aOR, adjusted odds ratio; CI, confidence interval.

Adjusted odds ratios for quitting all tobacco products are estimated by the logistic regression analysis.

^a^Age reference group: 20–39 years.

## DISCUSSION

We longitudinally investigated behavioral changes in tobacco use for 1 year before and during the COVID-19 pandemic. Our findings indicated a significant increase in the proportion of people who ceased using all tobacco products and conventional tobacco products during the pandemic, while no significant increase was observed in the proportion of people who ceased novel tobacco products. Among tobacco users, dual users showed a significant increase in tobacco cessation, whereas exclusive novel tobacco product users showed a significant decrease in cessation during the pandemic. We demonstrated a significant association between the intention to quit and tobacco cessation among dual users and exclusive conventional tobacco product users, while this association was not observed among exclusive novel tobacco product users.

Our study showed that dual users were less likely to achieve tobacco cessation before the COVID-19 pandemic; however, this probability increased significantly during the pandemic. This result is partially consistent with that of a previous report suggesting that dual use promotes nicotine dependence and makes it more difficult to quit tobacco.^15^^,^^16^ Conversely, some cigarette users are reported to start using novel tobacco products to quit smoking,^7^^,^^17^ so dual users who had previously been interested in quitting may have been more likely to quit successfully during the COVID-19 pandemic. Several studies suggest that cigarette smoking may serve as a potential risk factor for severe manifestations of COVID-19 pandemic.^18^^–^^20^ Furthermore, the pandemic has raised awareness of health issues and motivated a larger number of people to try to quit smoking.^11^^,^^21^^–^^26^ This increase in awareness may have inspired tobacco users to quit and contributed to the significant rise in the proportion of cessation of all tobacco products and conventional tobacco products observed in our study. The positive impact of the intention to quit among exclusive conventional tobacco product users and dual users in our study supports this hypothesis. On the other hand, there was no significant difference in the proportion of cessation among exclusive novel tobacco product users even during the COVID-19 pandemic. Furthermore, intention to quit had no significant impact on exclusive novel tobacco product users. There are some possible explanations for this trend. First, there is currently only a limited amount of information available about the COVID-19 pandemic and its relationship with novel tobacco products, leading to misunderstandings that novel tobacco products are less harmful than conventional tobacco products.^27^^–^^29^ During the COVID-19 pandemic, tobacco industries aggressively promoted HTPs, emphasizing that people can use HTPs more comfortably and safely at home.^5^^,^^16^^,^^30^ These tobacco industry campaigns undermined tobacco cessation initiatives by portraying HTP use as an easy “alternative” to breaking nicotine addiction and as socially acceptable during the COVID-19 pandemic.^27^^,^^30^ Those who intend to quit may opt for HTPs based on misinformation, leading to a lower chance of success in quitting.^29^ Second, HTPs may be preferred due to the increased time spent at home due to the COVID-19 pandemic because they produce relatively less smoke. Some may use HTPs as a means to evade smoke-free policies or for use in places where smoking is prohibited.^27^^,^^31^^,^^32^ Third, increased health awareness due to COVID-19 pandemic may have motivated smokers to choose HTPs because a previous study suggested that those who better understand the health consequences of tobacco use and those who express greater interest in quitting are more likely to choose ‘light’ cigarettes with the intention of reducing the health risks.^33^^–^^35^

While cigarette sales have declined over the years, sales of HTPs are on the rise in Japan; this trend was accelerated by the COVID-19 pandemic.^9^ In our study, the proportion of people who quit conventional tobacco products significantly increased during the pandemic, whereas the proportion of people who quit HTPs did not change. However, exclusive cigarette users were more likely to become dual users rather than exclusive HTP users both before and during the pandemic. This is inconsistent with the tobacco industry’s claim that switching from cigarettes to HTPs will reduce health hazards.^1^^,^^36^ Contrary to the tobacco industry’s claims that HTPs are a better alternative to cigarettes, they could hinder cessation among some individuals by prolonging or increasing addiction to nicotine.^27^^,^^29^ Given that HTPs do not bring health benefits and do not help tobacco cessation, HTPs should not be recommended for any purpose and should be included in a comprehensive approach to tobacco control.

This study has several limitations. First, the data were collected through internet surveys, which may have introduced selection bias towards participants with internet access. Second, since this study consisted of two different surveys with different purposes, the participants in each survey may have had different baseline characteristics, potentially affecting the generalizability of the study results. To address these limitations, the data were corrected using sampling probability weighting based on existing data from the Comprehensive Survey of Living Conditions. Third, the findings from this study, which was conducted in Japan, the world’s largest market for HTPs, may not be directly applicable to other countries due to differences in marketing strategies, regulations, and other environmental circumstances. Fourth, it was not possible to distinguish between e-cigarettes and HTPs because e-cigarettes are not as popular as HTPs and they are often confused in Japan. Finally, the self-reported nature of the survey may have resulted in recall bias or misreporting, although invalid responses were excluded from the analysis after conducting a response consistency check.

Despite these limitations, our study has several strengths. Previous studies were mostly cross-sectional with short observation periods and did not clearly distinguish the types of tobacco used. In contrast, this study used a longitudinal prospective design to observe changes in tobacco use status over 1 year before and during the COVID-19 pandemic. This allowed us to analyze the impact of COVID-19 pandemic on tobacco use status by the type of tobacco used.

### Conclusion

Cigarette and novel tobacco product use patterns varied in response to the COVID-19 pandemic. Our findings suggest that COVID-19 pandemic may have played a role in increasing cessation among those using conventional tobacco products. On the other hand, the COVID-19 pandemic did not have a positive effect on cessation of novel tobacco products. Promoting tobacco cessation is a current public health priority, and the COVID-19 pandemic may have provided opportunities for advancing tobacco control measures.

## SUPPLEMENTARY MATERIAL

eTable 1. Baseline characteristics of the participants (unweighted)

eTable 2. Changes of tobacco use status from the previous year (weighted)

eTable 3. Changes of tobacco use status from the previous year (unweighted)

eTable 4. Adjusted odds ratios for the pandemic group with pre-pandemic group as reference are estimated by the logistic regression analysis without adjustment

eFigure 1.

eFigure 2.
