# Supplementary material for: Impact of the COVID-19 Pandemic on Changes in Tobacco Use Behavior: A Longitudinal Cohort Study in Japan
Source: J Epidemiol. 2025 Jun 5;35(6):255–61. doi: 10.2188/jea.JE20240180 (PMC12066193; doi:10.2188/jea.JE20240180)
Supplement: Supplementary file 1 [file je-35-255-s001.pdf]

eTable 1. Baseline characteristics of the participants (unweighted)

	Group	Pre-pandemic		Pandemic		*P* value	SMD
Number		2196		2406			
Baseline research time	JASTIS 2019	2,196	(100.0)	0	(0.0)	<0.001	2.011
	JASTIS 2020	0	(0.0)	219	(9.1)		
	JACSIS 2020	0	(0.0)	2187	(90.9)		
Age, years	20s	128	(5.8)	238	(9.9)	<0.001	0.346
	30s	293	(13.3)	358	(14.9)		
	40s	584	(26.6)	613	(25.5)		
	50s	666	(30.3)	554	(23.0)		
	60s	446	(20.3)	395	(16.4)		
	70s	79	(3.6)	248	(10.3)		
Sex	Female	447	(20.4)	743	(30.9)	<0.001	0.243
BMI,[a] kg/m^2		22.95	[3.7]	22.82	[3.7]	0.232	0.035
Overweight or obesity[2]		561	(25.5)	569	(23.6)	0.144	0.044
Habitual alcohol intake		1,580	(71.9)	1,510	(62.8)	<0.001	0.197
Hypertension		445	(20.3)	466	(19.4)	0.469	0.022
Diabetes mellitus		186	(8.5)	197	(8.2)	0.770	0.010
Chronic respiratory diseases		137	(6.2)	127	(5.3)	0.182	0.041
Mental disorders		206	(9.4)	202	(8.4)	0.262	0.035
Self-reported health status[b]		2.6	[0.9]	2.51	[1.0]	0.003	0.088
Self-reported happiness[c]		4.74	[2.2]	4.52	[2.3]	0.001	0.099
Education	Over high school	1,561	(71.1)	1,582	(65.8)	<0.001	0.115
Working outside the home		1,833	(83.5)	1,880	(78.1)	<0.001	0.136
Annual household income	1st quartile	523	(23.8)	671	(27.9)	<0.001	0.180
	2nd quartile	416	(18.9)	499	(20.7)		
	3rd quartile	480	(21.9)	458	(19.0)		
	4th quartile	461	(21.0)	373	(15.5)		
	Unknown	316	(14.4)	405	(16.8)		
Living alone		427	(19.4)	517	(21.5)	0.093	0.051
Married		1,359	(61.9)	1,476	(61.3)	0.730	0.011

BMI, body mass index; SMD, standardized mean difference.

Data are shown with number and (percentage) or mean and [standard deviation]. Differences between the groups are evaluated by SMD. *P* values are estimated by Chi-square tests for categorical variables and by t-tests for continuous variables.

[a] Overweight or obesity is defined as 25 kg/m^2 or over in a BMI.

[b] Self-reported health status is evaluated by 5 rank scores: 1 (excellent), 2 (good), 3 (so so), 4 (bad), and 5 (awful).

[c] Self-reported happiness is evaluated by 10 rank scores: 1(awful) to 10 (excellent).

eTable 2. Changes of tobacco use status from the previous year (weighted)

	Group	Prepandemic			Pandemic			*P* value
		N	Participants[a]	%	N	Participants	%	
Quit all tobacco products		195	/1,920	10.2%	371	/2,681	13.8%	<0.001
Conventional tobacco	Quit	233	/1,628	14.3%	395	/2,122	18.6%	<0.001
	Started	51	/293	17.4%	71	/560	12.7%	0.077
Novel tobacco	Quit	294	/1,017	28.9%	318	/1,171	27.1%	0.558
	Started	80	/903	8.8%	180	/1,510	11.9%	0.117

[a] Participants indicates the number of eligible individuals whose use of each tobacco product may have varied.

eTable 3. Changes of tobacco use status from the previous year (unweighted)

	Group	Pre-pandemic			Pandemic			*P* value
		N	Participants[a]	%	N	Participants	%	
Quit all tobacco products		247	/2,196	11.2%	366	/2,406	15.2%	<0.001
Conventional tobacco	Quit	273	/1,862	14.7%	375	/1,863	20.1%	<0.001
	Start	47	/334	14.1%	70	/543	12.9%	0.691
Novel tobacco	Quit	348	/1,232	28.2%	315	/1,097	28.7%	0.839
	Start	103	/964	10.7%	146	/1,309	11.2%	0.775

[a] Participants indicate the number of eligible individuals whose use of each tobacco product may have varied.

eTable 4. Adjusted odds ratios for the pandemic group with pre-pandemic group as reference are estimated by the logistic regression analysis without adjustment

		Unweighted		
		aOR	95% CI	*P* value
Quit all tobacco products		1.42	(1.19–1.68)	<0.001
Cigarette	Quit	1.47	(1.24–1.74)	<0.001
	Start	0.90	(0.61–1.34)	0.618
Novel tobacco	Quit	1.02	(0.85–1.23)	0.803
	Start	1.05	(0.80–1.37)	0.724

aOR, adjusted odds ratio; CI, confidence interval.

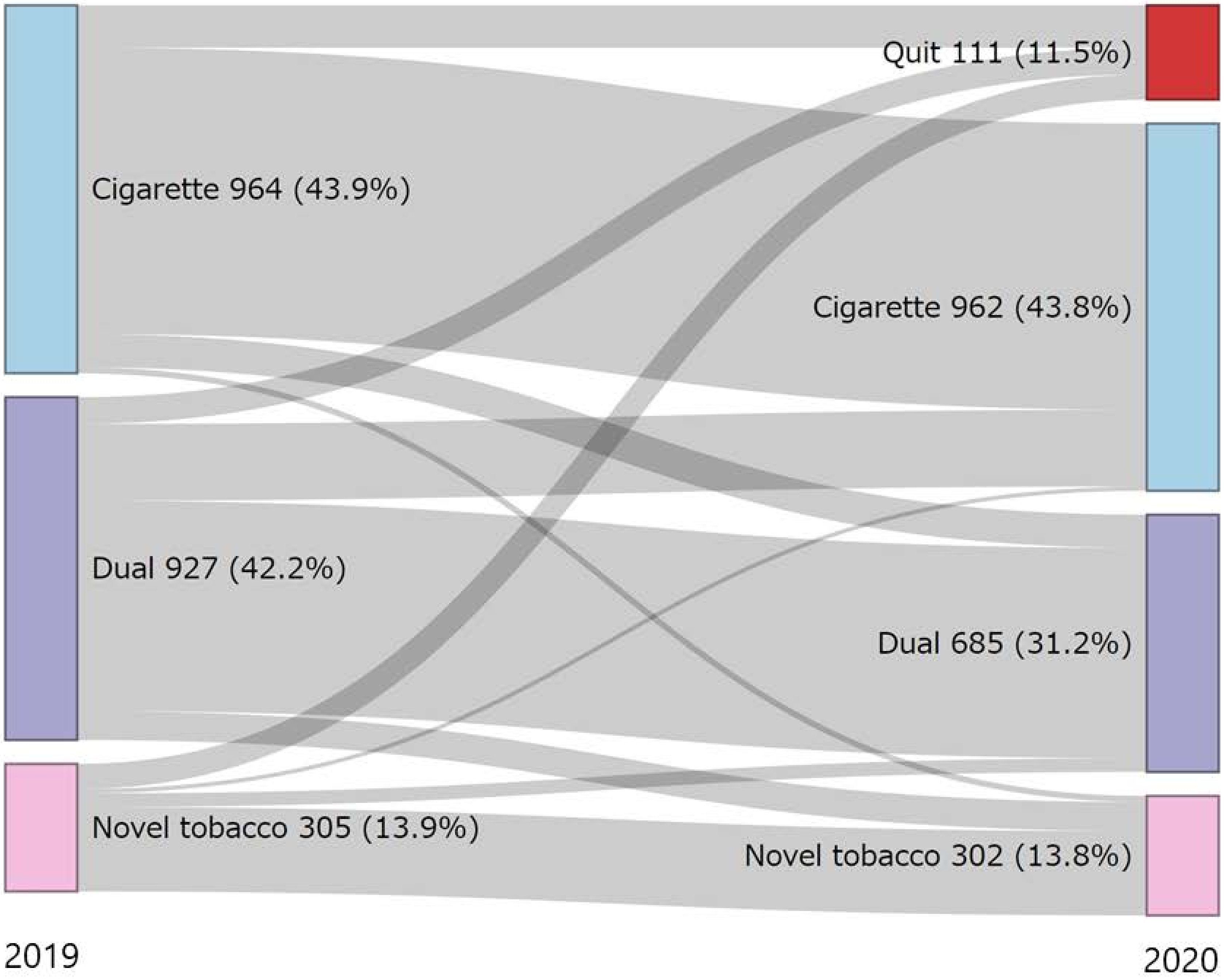
Cigarette 964 (43.9%)
Dual 927 (42.2%)
Novel tobacco 305 (13.9%)
2019
Quit 111 (11.5%)
Cigarette 962 (43.8%)
Dual 685 (31.2%)
Novel tobacco 302 (13.8%)
2020

eFigure 1

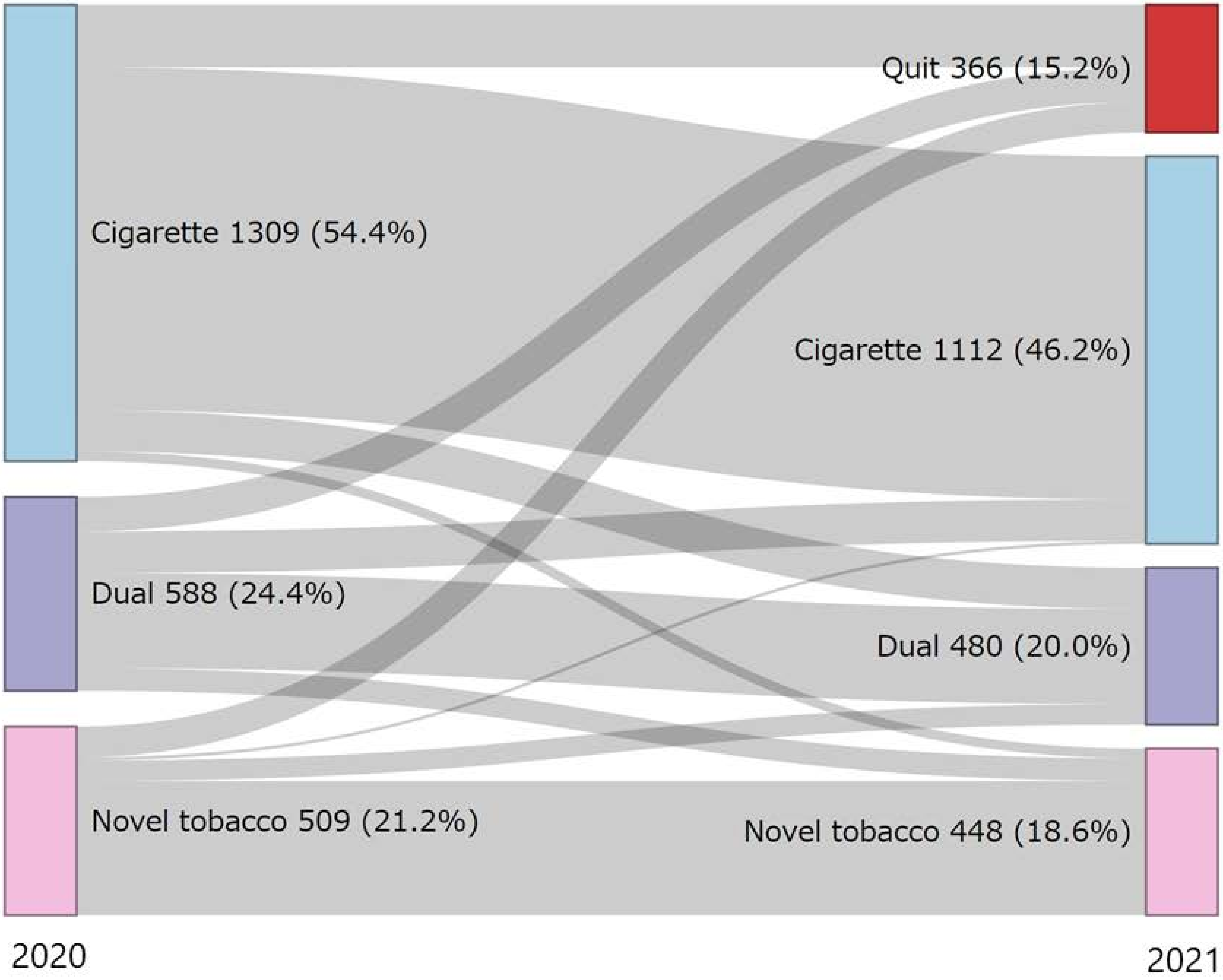
Cigarette 1309 (54.4%)
Dual 588 (24.4%)
Novel tobacco 509 (21.2%)
2020
Quit 366 (15.2%)
Cigarette 1112 (46.2%)
Dual 480 (20.0%)
Novel tobacco 448 (18.6%)
2021

eFigure 2
